# Advanced practice nursing: international gator nursing week

**DOI:** 10.1590/1980-220X-REEUSP-2024-0228en

**Published:** 2024-12-06

**Authors:** Beatriz Rosana Gonçalves de Oliveira Toso, Daiana Bonfim, Kelli Borges dos Santos, Neyson Pinheiro Freire, Mitzy Tannia Reichembach Danski, Priscilla Caroliny de Oliveira, Jeanne-Marie Rodrigues Stacciarini

**Affiliations:** 1 Universidade Estadual do Oeste do Paraná, Campus Cascavel, Centro de Ciências Biológicas e da Saúde, Curso de Enfermagem, Cascavel, PR, Brazil.; 2 Faculdade Israelita de Ciências da Saúde Albert Einstein, São Paulo, SP, Brazil.; 3 Universidade Federal de Juiz de Fora, Departamento de Enfermagem, Juiz de Fora, MG, Brazil.; 4 Conselho Federal de Enfermagem, Brasília, DF, Brazil.; 5 Universidade Federal do Paraná, Departamento de Enfermagem, Curitiba, PR, Brazil.; 6 University of Florida, College of Nursing, Gainesville, FL, USA.

**Keywords:** Enfermería de Práctica Avanzada, Enfermería, Educación en Enfermería, Aprendizaje, Advanced Practice Nursing, Nursing, Education, Nursing, Learning

## Abstract

**Objective:**

To describe the experience of the International Gator Nursing Week program focusing on advanced practice nurses’ (APNs) training and work in the United States of America.

**Method:**

An experience report on a one-week immersion course at the University of Florida College of Nursing, describing aspects related to APNs’ training structure and professional performance in mental healthcare services, Primary healthcare, pediatrics and hospital.

**Results:**

Advanced Practice Nursing (APN) refers to the clinical practice of nurses with specialized training and advanced healthcare skills, and these professionals are called APNs. The experience is described focusing on APNs’ educational training and professional performance in primary healthcare, mental health, pediatrics, advocacy for APN development, visits to primary healthcare services and research summits.

**Conclusion:**

The intensive experience in the program provided Brazilian nurses with the opportunity to learn about and participate in daily APN experiences in that country, expanding participants’ knowledge.

## RESUMEN

**Objetivo:**

Describir la experiencia del programa International Gator Nursing Week centrado en la formación y alcance del trabajo de los enfermeros de práctica avanzada (EPA) en los Estados Unidos de América.

**Método:**

Relato de experiencia de un curso de inmersión de una semana de duración en la Escuela de Enfermería de la University of Florida, describiendo aspectos relacionados con la estructura de formación y desempeño profesional del EPA en salud mental, Atención Primaria de Salud, pediatría y servicios hospitalarios.

**Resultados:**

La Enfermería de Práctica Avanzada (EPA) se refiere a la práctica clínica de enfermeros con formación especializada y habilidades avanzadas en el cuidado de la salud, con profesionales denominados EPAs. La experiencia se describe centrándose en la formación educativa y desempeño profesional del EPA en atención primaria, salud mental, pediatría, promoción para el desarrollo de la EPA, visitas a servicios de atención primaria y research summit.

**Conclusión:**

La experiencia intensiva en el programa brindó a los enfermeros brasileños la oportunidad de conocer y participar en las experiencias cotidianas de la EPA en ese país, ampliando los conocimientos de los participantes.

## INTRODUCTION

Advanced Practice Nursing (APN) has been developed in the United States of America (USA) since the 1960s^(1)^. This term encompasses a set of functions performed by nurses since then, and refers to the expansion of autonomy for the exercise of an expanded clinical practice based on appropriate training for this purpose^(2)^. APN emerged from the need to improve child health indicators in rural and hard-to-reach regions in the state of Colorado, USA, preparing nurses for primary healthcare for children and their families, with autonomy to diagnose health conditions and prescribe appropriate treatment, including medication, in addition to the skills that nurses already had, providing comprehensive and humanized care for prevention and health promotion^(3)^.

For clarification purposes, the definition of the terminology for each of the concepts on APN adopted in this article is as follows: a) APN refers to the area/field of knowledge; b) advanced practice nurses (APNs) is a comprehensive nomenclature used to designate the professional who works in this field of knowledge; c) a nurse practitioner is an APN who works in primary healthcare; d) a clinical nurse specialist is an APN who works in a specialty of knowledge^(1,2)^.

Currently, the USA has more than 385,000 APNs operating across all 50 states^(4)^. Autonomy for practice is not yet the same in all states, since each has its own state legislation for different sectors of society, including in the field of health. Thus, most states (27) allow APNs broad autonomy to act independently, with expanded clinical practice that includes advanced physical assessment (including physical examination findings (signs), patient complaints (symptoms), assessed for their impact on the likelihood of a disease), requesting diagnostic tests and assessing results, prescribing treatment, including medication. In other states, autonomy may be regulated or restricted, depending on local laws, in which APNs need to work in partnership with physicians to exercise their clinical practice^(5)^.

However, full practice authority (FPA) is a position of the American Association of Nurse Practitioners (AANP) to support the removal of regulatory barriers that prevent APNs from doing what they are trained to do and to advocate for APNs to practice within their full scope of practice. However, in those reduced or restricted practice states that require physician supervision, APNs have faced barriers to practice because professionals with the same education and training can practice freely in another state^(6)^. It is worth clarifying that, in the USA, the term “advanced practice registered nurse” (APRN) encompasses four different advanced practice roles: clinical nurse specialist (CNS); clinical nurse anesthetist (CNA); clinical nurse midwife (CNM); and nurse practitioner (NP)^(7)^.

In the USA, APNs acquire their training for this degree through a master’s degree and/or PhD, which include, in addition to theoretical subjects, a practical workload of at least 500 hours of clinical work in specific fields. After training, it is necessary to obtain accreditation from institutions with this purpose in order to obtain certification and, only then, to work in the role for which they obtained the degree^(8)^.

The first Doctor of Nursing Practice (DNP) program opened at the University of Kentucky College of Nursing (UKCON) in 2001^(9)^, but growth in offerings has been accelerating since then. There are currently 426 DNP programs in the USA, with an active enrollment of over 41,000 students and another 80 in the planning stages^(4)^.

Thus, this article aimed to describe the experience of the International Gator Nursing Week program with a focus on APNs’ training and work in the USA.

## METHOD

### Study Design

This is an experience report on the International Gator Nursing Week, promoted by the International Department of Nursing College, at the University of Florida (UF), USA.

### Local

UF is a public university located in Gainesville, with extension *campi* in cities such as Orlando and Jacksonville, and traces its origins back to 1853. It currently has 16 colleges, more than 150 research centers, and a student population of over 60,000. Since 2013, it has been designated a preeminent university.

### University of Florida College of Nursing

At UF, whose College of Nursing has been in operation since 1956, the master’s-level APN Education Program began in the mid-1970s, and in 2007, this training became exclusively at the PhD (DNP) level. Currently at UF, this training is offered in the following fields: 1) family care practice (primary healthcare); 2) gerontology (critical care - acute); 3) psychiatry and mental health; 4) pediatrics (primary or acute care or both).

### International Gator Nursing Week

The International Gator Nursing Week aims to characterize APN in the USA and provide opportunities for international partnerships with universities in Brazil and other Latin American countries. The course system was developed as an immersion course over a period of one week, from April 8 to 12, 2024, in Gainesville, Florida, USA, with theoretical classes and technical visits to healthcare services, totaling 40 hours. The course program covered content and visits to healthcare services, such as education/professional training, mental health, pediatrics, health advocacy, visits to healthcare services (hospital and rural clinic) and a “research summit” (participation/presentation at the research event and reports on improvements in the quality of services at the College of Nursing).

The course was organized and managed by Dr. Jeanne-Marie Stacciarini from UF. In 2024, 12 Brazilian nurses from various educational institutions participated, in addition to members of the Federal Council of Nursing (In Portuguese, *Conselho Federal de Enfermagem* (COFEN)), such as the *Universidade Estadual do Oeste do Paraná* (UNIOESTE), *Universidade Federal do Paraná* (UFPR), both in the state of Paraná, *Universidade CEUMA*, in the state of Maranhão, *Universidade Federal de Juiz de Fora* (UFJF), in the state of Minas Gerais, *Faculdade Israelita de Ciências da Saúde Albert Einstein* (FICSAE), in the state of São Paulo, *Pontíficia Universidade Católica* (PUC) and *Universidade Federal de Goiás* (UFG), both in the state of Goiás.

### Ethical Aspects

This manuscript, as it is an experience report, does not require the use of an Informed Consent Form (ICF) and the opinion of a Research Ethics Committee.

## RESULTS

### Experience Report

The International Gator Nursing Week program began in 2019, focusing on understanding and experiencing what APN is in the USA as well as facilitating international and research partnerships with different Latin American universities. The program initially included only professors from Brazilian universities, and was later expanded in partnership with COFEN and the Brazilian Ministry of Health, providing opportunities for integrating professionals from all Brazilian states. To date (April 2024), 70 Brazilian nurses have participated in the program.

The International Gator Nursing Week schedule varies slightly each year, but maintains its central purpose of demonstrating APN teaching and care by involving UF College of Nursing professors from different fields, students and graduates, and professionals in practice who present their experiences and work routines to Brazilian nurses. In 2024, APNs from the Florida U.S. Army Veterans Hospital participated.

In addition to presentations, the program includes a visit to the College of Nursing teaching hospital and clinic. The visit to the teaching hospital provides an insight into various hospital units and how APN serves as a leader in the units in which it operates as well as the role of APNs in hospital service when providing advanced practice care. The program also includes presentations by national leaders who demonstrate how AANP supports the expansion of the profession internationally and nationally, with support for education and policies that promote the profession and professionals in that country.

The program is being assessed, and in 2024, UF students, under the supervision of Dr. Jeanne-Marie Stacciarini, in partnership with a graduate of the *Universidade Federal Fluminense* College of Nursing, prepared the results of this assessment, which were presented at the Sigma Teta Tau congress in 2024 with the title “Global exchange advancing APRNs in Brazil”.

The experience of the 2024 International Gator Nursing Week will be presented by describing the aspects of the experience, according to authors’ perception, related to APN training by UF, APNs’ professional performance in mental healthcare services, primary healthcare, pediatrics and hospital care, advocacy, and research summit.

### Advanced Practice Nurse Training from the University of Florida

During International Gator Nursing Week, it was possible to learn about the main models of professional training for APNs in the USA, contextualizing the historical evolution of this training in the country. Moreover, each state has training models that respond to local needs, but they all seem to have in common a robust training for clinical and leadership skills.

The study presented and discussed differences between the master’s and PhD programs in advanced practice (DNP) and core competencies developed by the programs. The professor and director of the DNP program at UF shared the evolution of advanced practice programs, highlighting the major historical milestones and key points, which are described below:

DNP is a clinical PhD program with a strong emphasis on developing clinical skills and applied research in practice, and lasts three to four years. Thus, it is not focused on developing academic skills and research products, as is traditionally found in PhD programs (Chart 1). In DNP, research competency is related to the implementation of science to improve patient care, services, and health policies.

**Chart 1:** Comparison of the Doctor of Nursing Practice program and the PhD program presented at the International Gator Nursing Week. Florida, USA, 2024.

Aspects described	Doctor of Nursing Practice (DNP)	PhD
Centrality of the curriculum	Translate research evidence into nursing practice; Create healthcare policy (including budgets, financial management, leadership theory); Develop practical experience.	Research methodologies; Nursing research theories.
Prerequisites	Hold a Bachelor of Science in Nursing (BSN) or Master of Science in Nursing (MSN).	Some programs require an MSN, whereas others accept applicants with a BSN.
Credits/hours	Typically between 70–95 credits for those entering with a bachelor’s degree; Typically around 35 credits are required for students who have already obtained a master’s degree.	Approximately 60 credit hours for those with a master’s degree, including dissertation hours.
Clinical practice	Between 500 and 1,000 hours.	Minimum.
Search	Statistics and research theory for NP; Research methods and evidence-based practice.	Courses in quantitative methods, mixed methods, statistics I and II, advanced research; In-depth research projects guided by faculty.
Final project (thesis)	It often consists of a practice-focused quality improvement project with a presentation to stakeholders. Students present their work at the site where it was developed and at a clinical event.	Thesis that contributes substantially to knowledge production for nursing, in addition to the dissertation defense (board).

*Source*: Presented at International Gator Nursing Week.

During the first year of training, the courses are general to all DNP specialties: 1) theories and research for implementing APN; 2) physiology and pathophysiology for APN; 3) health promotion; 4) applied statistics; 5) scientific methods for the application of science; 6) advanced health assessment and diagnostic reasoning; 7) leadership in health systems; and 8) informatics applied to nursing. After this period, students take another year and a half with courses in the field of their specialty, such as specific diagnosis, adult health, family health (for primary healthcare APN).

The core competencies for APNs recommended by the American Association of Colleges of Nursing (AACN) are as follows: 1) knowledge for nursing practice; 2) person-centered care; 3) population health; 4) scholarship for the nursing discipline; 5) quality and safety; 6) interprofessional partnerships; 7) systems-based practice; 8) informatics and healthcare technologies; 9) professionalism; 10) personal, professional, and leadership development.

The theoretical content for DNP is taught in an asynchronous online format, and the assessment model is based on the development of theoretical, technical and clinical skills, management and leadership. DNP student assessment is carried out through simulated practical scenarios in person, and occurs virtually or in realistic simulation laboratories.

The total clinical workload for DNP is approximately 1,000 practical hours, of which 624 hours are spent providing direct patient care under the supervision of an APN or a physician in the same field. The choice for the physician is due to the insufficient number of APNs for all students. However, it is clarified that although the actions implemented by physicians and APNs are the same, the approach is quite different, as APNs have a holistic and comprehensive approach to nursing added to clinical practice.

The final project can be characterized as a quality improvement project, in which concepts of implementation science and quality management are applied. A traditional PhD thesis defense committee is not formed. The project is supervised and assessed by a professor who examines the relevance, social impact and expanded results for the service/community. Students must present their final project in the clinic or community where the work was carried out and in a clinical scientific event appropriate to the specialty, with studies relevant to the clinical context in which they are developed, producing local changes valued by different stakeholders.

### Training and Professional Performance of Advanced Practice Nurses in Mental Health

During the International Gator Nursing Week, the main models of professional training for APNs in psychiatry and mental health were presented by professors, who are APNs in mental health and described their professional trajectories and fields of activity.

The presentations emphasized a training centered on the four Ps (psychotherapy, pathophysiology, physical and psychiatric assessment, and psychopharmacology). DNP training in mental health offers general courses and five specific courses, including: 1) advanced psychiatric assessment/diagnosis; 2) psychopharmacology; 3) family therapy; 4) group therapy; and 5) final project. In addition to this, students complete approximately 780 clinical hours in a variety of settings serving patients with mental disorders.

APNs’ role and work in mental health are as a provider of specialized care, educator, and leader (working in several organizations and influencing health policies). Additionally, an APN from the veteran’s hospital also presented his professional role related to the system where he works as well as the challenges and successes of the profession.

Two APNs outlined their various roles, which include being a coordinator of psychiatric/mental healthcare (inpatient and outpatient), mental health educator (professor), milieu manager (hospital unit manager), manager, and provider of various psychological, drug, and alternative therapies.

In general, the presentations summarized that mental health APNs are: 1) a nurse; 2) an empathic professional (uses the self to therapeutically treat others); 3) a specialized professional; and 4) an advanced practice professional, with a minimum of a master’s degree to provide advanced person-centered care.

Although mental health APNs are highly regarded by other healthcare professionals and the general public, professional challenges remain real—health insurance companies are pushing for an emphasis on prescription medication. They also highlighted the high risk of burnout for APNs in mental health due to lack of time for the important role of psychotherapists. Ironically, but under US law, the APN who works at the Veterans Affairs hospital in Florida (considered a federal job) has full practice authority, and the other who works as a professor and practices at the UF clinic (considered a state job) does not have the same authority/autonomy, even though they are both practicing in the same state.

### Advanced Practice Nurse Training in Pediatrics

The International Gator Nursing Week in pediatrics aimed to provide Brazilian nurses with an understanding of the teaching-learning process for advanced practice competencies in pediatrics, through clinical simulation with high-performance mannequins and real actors. Thus, the program developed activities on how UF professors combine teaching and clinical practice in the hospital to care for newborns and hospitalized children, keeping professionals up to date and practicing with the best evidence.

The proposed activity included a technical visit to clinical practice simulation laboratories as well as a demonstration of an advanced clinical case simulating a surgical condition in a newborn. Some of the Brazilian visitors were arranged in the case application room and the other part in the control room. In the clinical case application room, the newborn mannequin was in a radiant heat crib accompanied by its mother (an actress representing the mother), when a nurse (a DNP student) arrived to assess the child and provide guidance to the mother. In the control room, the professor adjusted the mannequin’s complex functions according to the questions and the physical assessment performed by the nurse. At the end of simulated practice, everyone went to the debriefing room to listen to the professor’s feedback to the PhD student.

The learning objectives of the supervised simulation performed by a DNP student were to: demonstrate appropriate therapeutic communication strategies; perform a specific physical assessment for the infant with gastrointestinal disease; use physical assessment data to make decisions about care management; assess the impact of care decisions on patient clinical condition; and revise the management plan as needed.

The simulated case followed in the program was a 4-day-old male infant measuring 45 centimeters and weighing 2,780 grams. The problem described was vomiting since birth. In the simulated assessment, the infant was crying, tachypneic, hyperthermic, and tachycardic. On physical examination, the fontanelles were sunken and the abdomen was distended. The PhD student was advised that the infant was under her care and that she should take appropriate action.

After the simulated performance, with the consent of the student, the feedback she received from the professor was monitored, who commented on the case and considered the adopted conduct, guiding the student on the aspects she addressed. The professor used the SBAR (Situation, Background, Assessment, Recommendation) report in student assessment to comment on her performance.

It is worth noting that following the simulated practice to acquire clinical skills in newborn care brought clarity about APN, demonstrating its abilities and how this teaching-learning process can enable acquiring skills that had not yet been developed.

### Advocacy for Developing Advanced Practice Nursing

One of the goals of International Gator Nursing Week was to present AANP and its actions, mainly advocacy actions, to develop ANP in the USA.

The AANP president from the previous term (2021-2023) presented the organization’s work in representing nurses in favor of career advancement. AANP advocates for all NPs to provide accessible, person-centered, equitable, and high-quality healthcare to diverse communities through practice, education, advocacy, research, and leadership. AANP’s priorities were: serving as a public relations and public education agency for NPs; fighting to remove barriers and ensure full and direct access to NP services; offering a diverse selection of free, quality continuing education; helping NPs maximize their professional development and build their networks; conducting clinical outcomes research that supports health policy and practice; and providing easy access to the latest news and leading journals on APN.

This presentation demonstrated that AANP supports and promotes the role of APNs, who work to assist in career development and skill development, with opportunities for continuing education and participation in conferences organized by the association, providing access to peer-reviewed journals and professional publications, APN participation in research, and acting to shape the future of the profession through health policy.

AANP advocates for the role of APNs, provides discounts or reduced rates on continuing education (CE) and provides exclusive networking opportunities. AANP also supports NPs in all fields of practice and throughout their careers so that they can provide the best care to their patients. Furthermore, AANP connects NP groups that share common goals to advance knowledge and best practices in specific fields, such as acute care, pain management, cardiology, dermatology, and others.

### Visits to Primary Healthcare Services and Hospital Care

During International Gator Nursing Week, a visit was made to a primary healthcare clinic at the UF College of Nursing, called Archer Family Healthcare Clinic (AFHC), and hospital sectors at UF Health Shands Hospital. The purpose of the visits was to learn about the care process provided by APNs in care units.

### Primary Healthcare Unit

The experience took place in a rural town, Archer, located in Alachua County, Gainesville Metropolitan Region, in the state of Florida, USA. According to the 2020 census, Alachua had a population of 1,140 inhabitants. The visit was carried out at AFHC, which belongs to UF, and was carried out by the director, who, in addition to being a professor at the university, works as an NP (with a DNP degree in primary healthcare) at the clinic, and was attended by the team of other NPs from the clinic in demonstrating the unit to the Brazilian visitors.

At AFHC, healthcare for the local population is entirely provided by APNs (who, even in a state with reduced autonomy, diagnose health conditions and treat them, and have the authority to refer patients to other points in the network), with a total of four in primary healthcare and two nurses specialized in psychiatry and mental health. Primary healthcare APNs are qualified to assess and treat adult and pediatric patients with acute or chronic conditions. The most common cases are patients with diabetes, hypertension, viral and bacterial infections, allergies, dermatitis, and osteoporosis.

Visits were carried out in order to learn about scheduled nursing appointments, health screening, health education practices, and care delivery, with full autonomy for treatments, procedures, interventions, test requests, therapeutic decision-making, and disease prevention, control, and management actions. In addition, AFHC provides quality healthcare to its enrolled population through partnerships with the US healthcare system, focusing on the family. The clinic accepts most major health insurance plans, offers reduced rates to uninsured patients, and is included in the information and communications system managed by government health agencies, allowing for efficient continuity of care and treatment.

It should be emphasized NPs’ competency in carrying out different activities exclusive to APN, even in a state with reduced autonomy, such as nursing consultation with advanced clinical competency, including anamnesis and physical examination for advanced clinical investigation, requesting and analyzing laboratory and imaging tests, diagnosing diseases, making decisions for therapeutic conduct, performing complementary tests such as biopsies and prescribing medications. Thus, it was possible to perceive, in the APN practiced in the clinic, advanced assessment, clinical reasoning and diagnostic skills.

Clinical competency and the ability to care for and assess patients are essential, but contrary to the general conception, it was the NPs’ attitudes that consolidated the admiration for this role during the visit. Patient-centered clinical practice centered was observed upon appointment and advanced clinical investigation. Moreover, the constant presence of nurses in the community promotes patient trust and respect for NPs. At the same time that NPs discuss treatment and the conditions for carrying it out, they also welcome, guide, advise them based on the context in which patients live and refer them to other professionals within patients’ financial and economic possibilities. There is a real, positive and healthy bond and interaction between professional and patient.

The clinic’s geographic location, close to the community, makes it easier for residents to travel to obtain healthcare. This makes interventions feasible and reduces preventable complications. Appointments are scheduled and monitored by nurses who monitor patients with chronic diseases, such as diabetes and hypertension, promoting adequate control of these conditions. Health education and regular monitoring contribute to adherence to complication treatment and prevention.

Another positive impact was knowing the information system. The clinic is integrated with the health information system, and allows all therapeutic information, laboratory tests and patient diagnoses to be interconnected and available to registered professionals who provide care. Thus, it is clear that APNs’ clinical decision is always based on patient history. This makes it possible to observe the real situation of resource optimization, as unnecessary expenses are not repeated due to the lack of information, and treatment implementation occurs after analyzing which was most effective.

### Hospital Unit

The visit to UF Health Shands Hospital began in the pediatric cardiac Intensive Care Unit and then in the operating room. Both locations are staffed by APNs and clinical nurses. They and several other healthcare professionals work as a team. In general, APNs assess patients, request and interpret tests, write medication prescriptions, and provide advanced and highly complex care.

Visiting these services, at different levels of care, helped the group understand the role of APNs in healthcare services as well as the perception of the differences between APN and other nurses.

### Research Summit: Learning about Advanced Practice Nurses’ Research Projects

Research and innovation in healthcare were also highlighted during the immersion week. On the last day of activity, the “Nursing Research Summit 2024” took place, promoted and held at the UF College of Nursing. The event discussed innovations in the application of Artificial Intelligence (AI) and evidence-based mobile solutions in nursing care, with the theme “Artificial Intelligence and Healthcare Solutions in Nursing: Optimizing Patient Care”.

This event showcased scientific and quality improvement studies for nursing practice, and especially for APN, with several innovative topics, such as protective and risk factors in nursing and the training of new nurses in evidence-based practical research. A broad sample of recent studies was highlighted, revealing the impact and growing credibility of UF in nursing research. Collaboration among nurses, data scientists and health managers stood out as essential to develop solutions that align with the specific needs of nursing practice and patient care. The event also featured two discussion panels on nursing education and simulation and healthcare solutions using AI, in addition to the exhibition of 83 scientific and quality improvement studies in poster format.

These insights shared among professionals in the field expand our understanding of the impact of emerging technologies on nursing and also inspire continued reflection on how to integrate new tools ethically and effectively into professional practice.

## DISCUSSION

Although the American and Brazilian healthcare systems are quite different, one based on insurance and the other on a universal and social welfare system, this immersion provided an understanding of the role of NPs in that country, offering a visualization of how this professional (in Brazil) can receive training for advanced clinical care. Especially in primary healthcare, NPs make a difference in that country, promoting access to those who are left out of the system and being decisive in the care provided, recognized as primary healthcare providers in the community in which they work.

The NP care model was similar to that performed by Brazilian nurses in primary healthcare, specifically those trained in *lato sensu* graduate studies in family health, whose focus has been on prevention and health promotion, but also on comprehensive care, rehabilitation, leadership and management of complex cases in the care network and focusing on the social determination of the health-illness process of individuals and communities. However, what differentiates both professionals is expanded clinical practice, which guarantees nurses autonomy for clinical care, unlike the Brazilian reality, since nurses do not have the authority to: prescribe all medications that require a prescription, regardless of medical control; request medical laboratory, imaging and device tests (without being bound by protocols and care); decide on and indicate medical treatments and therapies; perform differential clinical diagnosis, side effects, disease staging, or advanced health assessments; be responsible for clinical care for a group (portfolio) of users; make referral and counter-referral of users in the network; and be recognized as the first point of contact in the health unit and by the care network^(2)^.

In the United States, NPs in primary healthcare account for 88% of primary healthcare providers and 73.3% in family health. They provide one billion patient visits per year and prescribe medications in every state. A growing general population, aging baby boomers, and the number of individuals living with chronic diseases will drive the need for robust access to primary healthcare. NPs are considered an effective solution for affordable, patient-centered healthcare^(5)^.

In Brazil, nursing accounts for 57% of the work in health. Among nursing professionals, 25% are nurses, and of these, 72% work in family health and 15% in primary healthcare^(10)^. APN training and recognition in Brazil can change the country’s health reality, influencing patient- and population-centered care, and public policies that improve access and resolution of care in this field of care. It is noteworthy that APNs would be better prepared to improve health systems and policies, as occurred in realities in which it was implemented^(11)^. APN training and adoption has been a global trend since the 2000s to address global health disparities^(12)^.

Consideration is given to encouraging the necessary change in the academic training of Brazilian nurses with an emphasis on clinical practice. To this end, colleges need to understand that advanced practice is achieved with advanced clinical knowledge and assume that investment in training is crucial^(13)^. It should be considered that APNs must have advanced reasoning and clinical assessment skills to make decisions based on evidence of effect and agile and accurate results. APN confirms that it is a fundamental strategy to transform the community’s health, and the experience revealed the importance of these professionals in the American scenario, with an impact on access and resolution of care, in addition to the strong role in health promotion and disease prevention.

## CONCLUSION

Brazilian professional participation in the immersion week at UF was an enriching experience, due to the convergence between the implementation of research, innovation and APN. It should be emphasized APNs’ potential to provide the right to health, especially to those in distant rural and remote urban areas. These professionals can impact the right to health, which is still very unequal in our country.
