# Supplementary material for: Advanced practice nursing: international gator nursing week
Source: Rev Esc Enferm USP. 2024 Dec 6;58:e20240228. doi: 10.1590/1980-220X-REEUSP-2024-0228en (PMC11649065; doi:10.1590/1980-220X-REEUSP-2024-0228en)
Supplement: Translation

# Translation: Prática avançada de enfermagem: international gator nursing week

**DOI:** 10.1590/1980-220X-REEUSP-2024-0228pt

**Authors:** Beatriz Rosana Gonçalves de Oliveira Toso, Daiana Bonfim, Kelli Borges dos Santos, Neyson Pinheiro Freire, Mitzy Tannia Reichembach Danski, Priscilla Caroliny de Oliveira, Jeanne-Marie Rodrigues Stacciarini

**Affiliations:** 1 Universidade Estadual do Oeste do Paraná, Campus Cascavel, Centro de Ciências Biológicas e da Saúde, Curso de Enfermagem, Cascavel, PR, Brasil.; 2 Faculdade Israelita de Ciências da Saúde Albert Einstein, São Paulo, SP, Brasil.; 3 Universidade Federal de Juiz de Fora, Departamento de Enfermagem, Juiz de Fora, MG, Brasil.; 4 Conselho Federal de Enfermagem, Brasília, DF, Brasil.; 5 Universidade Federal do Paraná, Departamento de Enfermagem, Curitiba, PR, Brasil.; 6 University of Florida, College of Nursing, Gainesville, FL, USA.

**Keywords:** Prática Avançada de Enfermagem, Enfermagem, Educação em Enfermagem, Aprendizagem

## Abstract

**Objetivo:**

Descrever a experiência do programa *International Gator Nursing Week* com enfoque na formação e o escopo de trabalho dos enfermeiros de prática avançada (EPAs) nos Estados Unidos da América.

**Método:**

Relato de experiência sobre curso de imersão de uma semana junto à Escola de Enfermagem da *University of Florida*, descrevendo os aspectos relacionados à estrutura formativa e atuação profissional do EPA em serviços de saúde mental, Atenção Primária à Saúde, pediatria e hospitalar.

**Resultados:**

A Prática Avançada de Enfermagem (PAE) refere-se à prática clínica de enfermeiros com formação especializada e habilidades avançadas de cuidados de saúde, sendo os profissionais denominados EPAs. Descreve-se a experiência enfocando na formação educacional e atuação profissional do EPA na atenção primária, na saúde mental, em pediatria, advocacia para o desenvolvimento da PAE, visitas aos serviços de atenção primária e *research summit*.

**Conclusão:**

A vivência intensiva no programa propiciou aos enfermeiros brasileiros a oportunidade de conhecer e participar de experiências de PAE cotidianas naquele país, ampliando o conhecimento dos participantes.

## INTRODUÇÃO

A Prática Avançada de Enfermagem (PAE) vem sendo desenvolvida nos Estados Unidos da América (EUA) desde os anos 1960^(1)^. Esta denominação abrange um conjunto de funções desempenhadas por enfermeiros desde então, e referese à ampliação da autonomia para o exercício de uma prática clínica ampliada a partir da formação apropriada para tal^(2)^. A PAE emergiu da necessidade de melhorar indicadores de saúde infantil em regiões rurais e de difícil acesso no estado do Colorado, EUA, preparando o enfermeiro para cuidados primários à criança e suas famílias, com autonomia para o diagnóstico de condições de saúde e a prescrição da terapêutica apropriada, inclusive medicamentosa, para além das habilidades que o enfermeiro já possuía, realizando um cuidado integral e humanizado de prevenção e promoção da saúde^(3)^.

A título de esclarecimento, apresenta-se a definição da terminologia para cada um dos conceitos sobre PAE adotados neste artigo: a) PAE refere-se à área/campo de conhecimento; b) enfermeiros de prática avançada (EPAs) é uma nomenclatura abrangente utilizada para designar o profissional que atua nesse campo do conhecimento; c) *nurse practitioner é* o EPA que atua na atenção primária (enfermeiro clínico generalista); d) *clinical nurse specialist é* o EPA que atua em uma especialidade do conhecimento (enfermeiro clínico especialista)^(1,2)^.

Atualmente, os EUA têm mais de 385 mil EPAs atuando nos 50 estados americanos^(4)^. A autonomia para a prática ainda não é igual em todos os estados, uma vez que cada um tem legislação estatal própria para os setores da sociedade, inclusive na área da saúde. Dessa forma, a maioria dos estados (27) possibilita aos EPAs ampla autonomia de atuação independente, com prática clínica ampliada que inclui a avaliação física avançada (inclui os achados do exame físico (sinais), as queixas do paciente (sintomas), avaliados quanto ao seu impacto na probabilidade de uma doença), solicitação de exames diagnósticos e avaliação dos resultados, prescrição da terapêutica, inclusive medicamentosa. Nos demais estados, a autonomia pode ser regulada ou restrita, a depender das leis locais, em que o EPA necessita trabalhar de forma conveniada ao médico para exercer sua prática clínica^(5)^.

Todavia, a autoridade de prática plena (*full practice authority* (FPA)) é uma posição da Associação Americana de Enfermeiros de Prática Avançada (*American Association of Nurse Practitioner* (AANP)) para apoiar a remoção de barreiras regulatórias, as quais impedem o EPA de fazer o que foram educados para fazer, defendendo que os EPAs pratiquem dentro de seu escopo completo de atuação. Entretanto, naqueles estados de prática reduzida ou restrita, cujo requisito é a supervisão médica, os EPAs têm enfrentado barreiras para sua atuação, pois profissionais com a mesma educação e treinamento podem praticar livremente em outro estado^(6)^. Vale esclarecer que, nos EUA, o termo “enfermeiro de prática avançada registrado” (*advanced practice registered nurse* (APRN)) engloba quatro diferentes atuações de prática avançada: enfermeiro clínico especialista (*clinical nurse specialist* (CNS)); enfermeiro anestesista (*clinical nurse anesthetist* (CNA)); enfermeiro obstetra (*clinical nurse midwife* (CNM)); e enfermeiro clínico generalista (*nurse practitioner* (NP))^(7)^.

Nos EUA, o EPA adquire sua formação para essa titulação por meio do mestrado e/ou doutorado profissional, os quais incluem, além das disciplinas teóricas, uma carga horária prática de no mínimo 500 horas de atuação de clínica em áreas especificas. Após a formação, é preciso realizar a acreditação em instituições com esta finalidade para obter a certificação e, somente então, atuar na função para a qual obteve a titulação^(8)^.

O primeiro programa Doutor em Prática de Enfermagem (DNP) foi aberto na Escola de Enfermagem da Universidade de Kentucky (UKCON) em 2001^(9)^, mas o crescimento da oferta tem aumentado muito desde então. Atualmente, existem 426 programas DNP nos EUA, com uma matrícula ativa de mais de 41.000 alunos e outros 80 em fase de planejamento^(4)^.

Assim, este artigo tem como objetivo descrever a experiência do programa *International Gator Nursing Week* com enfoque na formação e no escopo de trabalho dos EPAs nos EUA.

## MÉTODO

### Desenho do Estudo

Trata-se de relato de experiência sobre a International Gator Nursing Week, promovido pelo International Department da Nursing College, na University of Florida (UF), EUA.

### Local

A UF é uma universidade pública localizada em Gainesville, com campi de extensão em cidades tais como Orlando e Jacksonville, com sua origem traçada em 1853. Atualmente, tem 16 faculdades, mais de 150 centros de pesquisa e uma população de mais de 60.000 alunos. Desde 2013, recebeu a designação de uma universidade preeminente.

Faculdade de enfermagem na University of Florida

Na UF, cuja Escola de Enfermagem está em atividade desde 1956, o Programa de Educação de PAE com mestrados teve início em meados dos anos de 1970, e em 2007, essa formação passou a ser exclusivamente a nível de doutorado (DNP). Atualmente na UF, essa formação é oferecida nas seguintes áreas: 1) prática de atenção à família (atenção primária); 2) gerontologia (cuidados críticos - agudo); 3) psiquiatria e saúde mental; 4) pediatria (atenção primária ou aguda ou ambos).

### International Gator Nursing Week

A International Gator Nursing Week tem como objetivo caracterizar a PAE nos EUA e oportunizar parcerias internacionais com universidades brasileiras e de outros países da América Latina. A sistemática desenvolvida pelo curso foi a de imersão, no período de uma semana, de 08 a 12 de abril de 2024, em Gainesville, estado da Flórida, EUA, com aulas teóricas e visitas técnicas em serviços de saúde, totalizando 40 horas. A programação do curso abrangeu conteúdos e visitas a serviços de saúde, como educação/formação profissional, saúde mental, pediatria, advocacia em saúde, visitas aos serviços de saúde (hospital e clínica rural) e “research summit” (participação/apresentação no evento de pesquisa e relatos de melhoria da qualidade dos serviços da Escola de Enfermagem).

O curso foi organizado e gerenciado pela Prof^a^. Dr^a^. Jeanne-Marie Stacciarini da UF. No ano de 2024, participaram 12 enfermeiros brasileiros de diversas instituições de ensino, além de membros do Conselho Federal de Enfermagem (COFEN), tais como Universidade Estadual do Oeste do Paraná (UNIOESTE), Universidade Federal do Paraná (UFPR), ambas no estado do Paraná, Universidade CEUMA, no estado do Maranhão, Universidade Federal de Juiz de Fora (UFJF), no estado de Minas Gerais, Faculdade Israelita de Ciências da Saúde Albert Einstein (FICSAE), no estado de São Paulo, Pontíficia Universidade Católica (PUC) e Universidade Federal de Goiás (UFG), ambas no estado de Goiás.

### Aspectos Éticos

O manuscrito, por sua característica metodológica de relato de experiência, dispensa o uso do Termo de Consentimento Livre e Esclarecido (TCLE) e parecer de Comitê de Ética em Pesquisa com Seres Humanos.

## RESULTADOS

### Relato da Experiência

O programa *International Gator Nursing Week* teve início em 2019, com o enfoque na compreensão e experiência sobre o que é a PAE nos EUA, bem como viabilizar parcerias internacionais e de pesquisa com diferentes universidades latino-americanas. O programa, inicialmente, incluiu somente professores de universidades brasileiras, sendo expandido posteriormente, em parceria com COFEN e Ministério da Saúde brasileiro, oportunizando a integração de profissionais de todos os estados do Brasil. Até o momento (abril de 2024), 70 enfermeiros brasileiros já participaram do programa.

A programação do *International Gator Nursing Week* apresenta pequenas variações anualmente, mas mantém a proposta central de demonstrar o ensino e a assistência do EPA envolvendo professores de diferentes áreas, alunos e egressos da Escola de Enfermagem da UF e profissionais da prática que apresentam suas experiências e rotinas de trabalho aos enfermeiros brasileiros. No ano de 2024, foi incluída a participação de EPAs do hospital dos veteranos do exército americano da Flórida.

Além de apresentações, o programa inclui uma visita no hospital escola e na clínica da Escola de Enfermagem. A visita no hospital escola auxilia no conhecimento de várias unidades hospitalares e como o EPA atua na liderança das unidades em que trabalha, além da atuação do EPA no serviço hospitalar na oferta de cuidados de prática avançada. O programa também inclui apresentação de líderes, a nível nacional, que demonstram como a AANP apoia a expansão da profissão internacionalmente e nacionalmente, com suporte de educação e políticas que promovem a profissão e os profissionais daquele país.

O programa está sendo avaliado, e em 2024, alunas da UF, sob supervisão da Prof^a^. Dr^a^. Jeanne-Marie Stacciarini, em parceria com uma egressa da Escola de Enfermagem da Universidade Federal Fluminense, elaboraram o resultado dessa avaliação, que foi apresentado no congresso da *Sigma Teta Tau*, em 2024, com o título de “*Global exchange advancing APRNs in Brazil”*.

A experiência do *International Gator Nursing Week* de 2024 será apresentada descrevendo os aspectos da experiência, conforme a percepção dos autores, relacionados à formação do EPA pela UF, atuação profissional do EPA em serviços de saúde mental, Atenção Primária à Saúde, pediatria e atenção hospitalar, *advocacy*, e *research summit*.

### Formação do Enfermeiro de Prática Avançada pela University of Florida

Durante o *International Gator Nursing Week*, foi possível conhecer os principais modelos de formação profissional de EPAs nos EUA, contextualizando a evolução histórica dessa formação no país. Além disso, cada estado possui modelos de formação que respondem às necessidades locais, mas todos parecem ter em comum uma formação robusta para as competências clínicas e de liderança.

Foram apresentadas e discutidas as diferenças entre os currículos de mestrado e doutorado em prática avançada (DNP) e as competências essenciais desenvolvidas pelos programas. A professora diretora do programa DNP, na UF, compartilhou a evolução dos cursos de formação em prática avançada, destacando os principais marcos históricos e pontos-chave, que são descritos a seguir:

O DNP é um doutorado clínico com grande destaque para o desenvolvimento de competências clínicas e de pesquisa aplicada à prática, com duração de três a quatro anos. Assim, não está focado em desenvolvimento de competências e produtos de pesquisa acadêmica, como tradicionalmente encontrado em programas de PhD (Quadro 1). No DNP, a competência de pesquisa está relacionada à implementação da ciência para melhoria da atenção ao paciente, dos serviços e das políticas de saúde.

**Quadro 1:** Comparação entre o programa Doutor em Prática de Enfermagem e o programa de PhD apresentado no *International Gator Nursing Week*. Flórida, EUA, 2024.

Aspectos descritos	Doutor em Prática de Enfermagem (DNP)	PhD
Centralidade do currículo	Traduzir evidências de pesquisa para a prática de enfermagem; Criar política de cuidados de saúde (incluindo orçamentos, gestão financeira, teoria de liderança); Desenvolver experiência prática.	Metodologias de pesquisa; Teorias de pesquisa em enfermagem.
Pré-requisitos	Possuir bacharelado em enfermagem (BSN) ou mestrado em enfermagem (MSN).	Alguns programas exigem MSN, enquanto que outros aceitam candidatos com BSN.
Créditos/horas	Geralmente entre 70–95 créditos para quem ingressa com um diploma de bacharel; Normalmente são necessários cerca de 35 créditos para alunos que já tenham obtido o título de mestre.	Cerca de 60 horas de crédito para quem tem mestrado, incluindo horas de dissertação.
Prática clínica	Entre 500 e 1.000 horas.	Mínimo.
Pesquisa	Estatística e teoria de pesquisa para NP; Métodos de pesquisa e prática baseada em evidência.	Disciplinas de métodos quantitativos, métodos mistos, estatística I e II, pesquisa avançada; Projetos de pesquisa aprofundados e orientados pelo corpo docente.
Projeto final (tese)	Muitas vezes consiste em um projeto de melhoria da qualidade focado na prática com uma apresentação às partes interessadas. O aluno apresenta seu trabalho no local que foi desenvolvido e em evento voltado para a clínica.	Tese que contribui substancialmente para a produção de conhecimento para a área da enfermagem, além de defesa de dissertação (banca).

Fonte: apresentado na *International Gator Nursing Week*.

Durante o primeiro ano de formação, as disciplinas são gerais para todas as especialidades do DNP: 1) teorias e pesquisa para implementação da PAE; 2) fisiologia e fisiopatologia para a PAE; 3) promoção da saúde; 4) estatística aplicada; 5) métodos científicos para a aplicação da ciência; 6) avaliação de saúde a nível avançado e raciocínio diagnóstico; 7) liderança nos sistemas de saúde; e 8) informática aplicada à enfermagem. Após esse período, o aluno cursa mais um ano e meio com disciplinas na área da sua especialidade, tais como diagnóstico específico, saúde do adulto, saúde da família (para o EPA da atenção primária).

As competências essenciais para o EPA recomendadas pela Associação Americana de Faculdades de Enfermagem (*American Association of Colleges of Nursing* (AACN)) são as seguintes: 1) conhecimento avançado para a prática de enfermagem; 2) cuidado centrado na pessoa; 3) saúde populacional; 4) conhecimento científico na área específica; 5) qualidade e segurança do cuidado; 6) trabalho em equipe e relações interdisciplinares; 7) prática centrada nos sistemas; 8) informática e tecnologia; 9) ética e profissionalismo; 10) desenvolvimento pessoal e profissional para liderança.

O conteúdo teórico para o DNP é ministrado na modalidade *online* assíncrona, e o modelo de avaliação é baseado no desenvolvimento de competências teóricas, técnicas e clínicas, de gestão e liderança. A avaliação do estudante de DNP é realizada por meio de cenários simulados da prática de forma presencial, e ocorrem virtualmente ou nos laboratórios de simulação realística.

A carga horária clínica total do DNP é de aproximadamente 1.000 horas práticas, sendo que 624 horas são de prática assistencial direta ao paciente sob a supervisão de um enfermeiro certificado em prática avançada ou médico da mesma área de atuação. A escolha pelo médico se dá devido ao número insuficiente de EPA para todos os alunos. Todavia, é esclarecido que embora as ações implementadas pelo médico e EPA sejam as mesmas, a abordagem é bastante diferenciada, pois o EPA tem a abordagem holística e integral da enfermagem acrescida à prática clínica.

O projeto final de curso pode ser caracterizado como um projeto de melhoria da qualidade, no qual são aplicados conceitos de ciência da implementação e de gestão da qualidade. Não é constituída uma banca de defesa tradicional da tese de doutorado. O projeto é supervisionado e avaliado por um professor que examina a pertinência, o impacto social e os resultados ampliados para o serviço/comunidade. O aluno tem que apresentar seu projeto final na clínica ou na comunidade onde o trabalho foi realizado e em um evento de cunho clínico científico apropriado para a especialidade, com produções relevantes ao contexto clínico onde são desenvolvidos, produzindo mudanças locais valorizadas por diferentes *stakeholders*.

### Formação e Atuação Profissional do Enfermeiro de Prática Avançada em Saúde Mental

Durante o *International Gator Nursing Week*, foram apresentados os principais modelos de formação profissional de EPAs na área da psiquiatria e saúde mental pelos professores, os quais são EPAs em saúde mental e descreveram suas trajetórias profissionais e áreas de atuação.

As apresentações ressaltaram a formação centrada nos quatro Ps (*psychoterapy, pathophisiology, physical and psychiatric assessment, and psychopharmacology* (em português, psicoterapia, fisiopatologia, avaliação física e psiquiátrica, e psicofarmacologia)). A formação do DNP em saúde mental oferece disciplinas gerais e cinco específicas como: 1) avalição psiquiátrica avançada/diagnóstico; 2) psicofarmacologia; 3) terapia de família; 4) terapia de grupo; e 5) projeto final. Além disso, os alunos completam aproximadamente 780 horas clínicas, em diferentes serviços de atendimento aos pacientes com transtornos mentais.

A atuação e o trabalho para o EPA em saúde mental são como provedor de cuidados especializados, educador e de liderança (atuando em diversas organizações e influenciando políticas de saúde). Adicionalmente, um EPA do hospital dos veteranos também apresentou o seu papel profissional relacionado ao sistema onde trabalha, assim como os desafios e sucessos da profissão.

Dois EPAs delinearam suas diversas funções, as quais abrangem ser coordenador do cuidado psiquiátrico/saúde mental (a nível hospitalar e ambulatorial), educador em saúde mental (professor), gerente de *milieu* (gerente da unidade hospitalar), gestor, e provedor de diversas terapias psicológicas, medicamentosas e alternativas.

Em geral, as apresentações sintetizaram que o EPA de saúde mental é: 1) um enfermeiro; 2) um profissional empático (usa o *self* para tratar terapeuticamente o outro); 3) um profissional especializado; e 4) um profissional de práticas avançadas, com uma formação mínima de mestrado para prestar cuidados avançados centrado na pessoa.

Embora o EPA da área da saúde mental tenha alto reconhecimento de outros profissionais de saúde e da população em geral, os desafios profissionais ainda são reais - os seguros de saúde pressionam para uma ênfase nas prescrições de medicamentos. Faltando tempo para o importante papel de psicoterapeuta, ressaltaram também o alto risco de *burnout* para o EPA em saúde mental. Ironicamente, mas seguindo a leis dos EUA, o EPA que trabalha no hospital dos veteranos no estado da Flórida (considerado trabalho a nível federal) tem autoridade de prática plena, e o outro que trabalha como professor e atende na clínica da UF (considerado nível estadual) não tem a mesma autoridade/autonomia, embora estejam ambos praticando no mesmo estado.

### Formação do Enfermeiro de Prática Avançada em Pediatria

O *International Gator Nursing Week* em relação à pediatria teve como objetivo que os enfermeiros brasileiros pudessem conhecer o processo de ensino-aprendizagem por competências de práticas avançadas na pediatria, por meio da simulação clínica com manequins de alta performance e atores reais. Assim, o programa desenvolveu atividades sobre como o professor da UF combina ensino e prática clínica no hospital no cuidado a neonatos e crianças hospitalizadas e mantém o profissional atualizado e praticando com as melhores evidências.

A atividade proposta incluiu a visita técnica aos laboratórios de simulação de prática clínica, bem como a demonstração de um caso clínico avançado, simulando uma condição cirúrgica em neonato. Uma parte dos visitantes brasileiros foi distribuída na sala de aplicação do caso, e a outra parte, na sala de controle. Na sala de aplicação do caso clínico, o manequim do recém-nascido estava em berço de calor radiante acompanhado de sua mãe (atriz representando a mãe), quando a enfermeira (aluna de DNP) chega para avaliar a criança e orientar a mãe. Na sala de controle, o professor foi ajustando as complexas funções do manequim de acordo com as perguntas e a avaliação física realizada pela enfermeira. Ao final da prática simulada, todos dirigiram-se à sala de *debriefing* para acompanhar o *feedback* do professor para a doutoranda.

Os objetivos de aprendizagem da simulação acompanhada, desempenhada por estudante de DNP, foram: demonstrar estratégias de comunicação terapêutica apropriadas; realizar a avaliação física específica para o neonato com doença gastrointestinal; utilizar os dados da avaliação física para tomada de decisão sobre o manejo do cuidado; avaliar o impacto das decisões sobre o cuidado na condição clínica do paciente; e revisar o plano de manejo de acordo com as necessidades.

O caso simulado acompanhado no programa foi de um neonato de 4 dias, do sexo masculino, com 45 centímetros e pesando 2.780 gramas. O problema descrito foi de êmese desde o nascimento. Na avaliação simulada, o neonato se apresentava choroso, taquipneico, hipertérmico e taquicárdico. Ao exame físico, as fontanelas estavam afundadas, e o abdômen, distendido. A aluna de doutorado recebeu a orientação de que o neonato estava sob seus cuidados e que ela deveria tomar a conduta apropriada.

Após a performance simulada, acompanhou-se, com permissão da aluna, o *feedback* que recebeu do professor, o qual comentou o caso e ponderou sobre as condutas adotadas, orientando a aluna sobre os aspectos que esta abordou. O professor utilizou o SBAR (*Situation, Background, Assessment, Recommendation* (em português, Situação, Informações Prévias, Avaliação, Recomendações)) *report* na avaliação da aluna para comentar sobre sua performance.

Destaca-se que acompanhar a prática simulada para aquisição de competências clínicas no cuidado ao neonato trouxe clareza sobre a PAE, demonstrando suas habilidades e como esse processo de ensino-aprendizagem pode possibilitar a aquisição de competências até então ainda não desenvolvidas.

### Advocacy para o Desenvolvimento da Prática Avançada de Enfermagem

Um dos objetivos do *International Gator Nursing Week* foi apresentar a AANP e suas ações, principalmente de *advocacy*, no desenvolvimento da PAE nos EUA.

A presidente da AANP da gestão anterior (2021-2023) apresentou a atuação da entidade na representação dos enfermeiros em prol dos avanços na carreira. A AANP promove *advocacy* para que todos os NPs possam oferecer cuidados de saúde acessíveis, centrados na pessoa, equitativos e de elevada qualidade para diversas comunidades por meio da prática, educação, *advocacy*, pesquisa e liderança. Apresentou como prioridades da AANP: atuar como relações públicas e promover a educação do público sobre NPs; lutar para remover barreiras e garantir o acesso total e direto aos serviços de NP; oferecer uma seleção diversificada de educação continuada gratuita e de qualidade; ajudar os NPs a maximizar seu desenvolvimento profissional e construir suas redes; conduzir pesquisas de resultados clínicos que apoiem a política e a prática de saúde; e fornecer acesso fácil às últimas notícias e aos principais periódicos sobre a PAE.

Esta apresentação demonstrou que a AANP apoia e promove o papel do EPA, que atua para auxiliar no desenvolvimento da carreira e aprimoramento de habilidades, com oportunidades de educação continuada e participação nas conferências organizadas pela associação, possibilitando o acesso a periódicos revisados por pares e publicações profissionais, a participação dos EPA em pesquisas, além de atuar para moldar o futuro da profissão mediante as políticas de saúde.

A AANP atua por meio da *advocacy* em prol da função de PAE, promovendo descontos ou taxas reduzidas em educação continuada (EC) e oportunidades exclusivas de *networking*. A ANNP também apoia o NP em todas as áreas de prática e ao longo de sua carreira, para que o profissional possa oferecer o melhor atendimento aos seus pacientes. Além disso, a AANP conecta os grupos de NP que compartilham objetivos comuns para o avanço do conhecimento e das melhores práticas em áreas específicas, como de cuidados agudos, controle da dor, cardiologia, dermatologia, entre outras.

### Visitas aos Serviços de Atenção Primária à Saúde e Atenção Hospitalar

Durante a *International Gator Nursing Week*, foi realizada atividade de visitação em uma clínica de atenção primária da faculdade enfermagem da UF intitulada *Archer Family Heath Care Clinic* (AFHC) e setores hospitalares da UF *Health Shands Hospital*. O objetivo das visitas foi conhecer o processo de assistência prestado nas unidades assistenciais por EPAs.

### Unidade de Atenção Primária à Saúde

A experiência se deu em uma cidade rural, Archer, localizada no condado de Alachua, Região Metropolitana de Gainesville, no estado da Flórida, EUA. De acordo com o censo de 2020, Alachua tinha uma população de 1.140 habitantes. A visita foi realizada na AFHC, pertencente à UF, e conduzida pela diretora, que além de professora da universidade, atua como NP (com a formação em DNP em atenção primária) na clínica, e contou com a participação da equipe de outros NPs da clínica na demonstração da unidade aos visitantes brasileiros.

Na AFHC, a atenção à saúde para a população local é integralmente executada por EPAs (os quais, mesmo em um estado com autonomia reduzida, realizam o diagnóstico de condições de saúde, seu tratamento, possuindo autoridade para referenciar pacientes para outros pontos da rede), com um total de quatro na atenção primária e dois enfermeiros especialistas em psiquiatria e saúde mental. Os EPAs da atenção primária estão habilitados para avaliar e tratar pacientes adultos e crianças com condições agudas ou crônicas. Os casos mais comuns são de pacientes com diabetes, hipertensão, infecções virais e bacterianas, alergias, dermatites, e osteoporoses.

O objetivo da visita foi conhecer as consultas de enfermagem agendadas, a triagem de saúde, a prática da educação em saúde e a implementação do cuidado, com autonomia total para tratamentos, procedimentos, intervenções, solicitação de exames, tomada de decisão terapêutica, ações de prevenção, controle e manejo de doenças. Além disso, a AFHC oferece cuidados de saúde de qualidade à sua população adscrita, por meio de parcerias com o sistema de saúde dos EUA, atendendo com enfoque na família. A clínica aceita a maioria dos principais planos de seguro de saúde, oferece taxas reduzidas a pacientes sem seguro e está incluída no sistema de informações e comunicações, gerenciado pelos órgãos governamentais de saúde, permitindo continuidade dos cuidados e tratamentos de forma eficiente.

Destaca-se a competência da NP em realizar diferentes atividades exclusivas da PAE, mesmo em estado com autonomia reduzida, tais como consulta de enfermagem com competência clínica avançada, incluindo anamnese e exame físico para investigação clínica avançada, solicitação e análise de exames laboratoriais e de imagem, diagnóstico de doenças, tomada de decisão para conduta terapêutica, realização de exames complementares como biópsia e prescrição de medicamentos. Dessa forma, foi possível perceber, na PAE praticada na clínica, as habilidades de avaliação, de raciocínio clínico e de diagnóstico avançados.

A competência clínica e a habilidade em cuidar e avaliar pacientes são essenciais, mas contrariando a concepção geral, foram as atitudes do EPA que consolidaram a admiração por essa função durante a visita. Após o atendimento e investigação clínica avançada, percebeu-se a prática clínica centrada no paciente. Além disso, a presença constante dos enfermeiros na comunidade promove a confiança e o respeito dos pacientes pelo NP. Ao mesmo tempo que o EPA discute a terapêutica e condições de realizá-la, ele também acolhe, orienta, aconselha com base no contexto que o paciente vive e encaminha a outros profissionais dentro das possibilidades financeiras e econômicas do paciente. Há um vínculo e interação real, positiva e saudável entre profissional e paciente.

A localização geográfica da clínica, próxima à comunidade, facilita o deslocamento dos moradores para obter cuidados de saúde. Isso viabiliza as intervenções e reduz complicações evitáveis. O agendamento e o controle dos atendimentos são realizados por enfermeiros que monitoram os pacientes com doenças crônicas, como diabetes e hipertensão, promovendo o controle adequado dessas condições. A educação em saúde e o acompanhamento regular contribuem para a adesão ao tratamento e prevenção de complicações.

Outro impacto positivo foi conhecer o sistema de informação. A clínica é integrada ao sistema de informação de saúde, e permite que todas as informações terapêuticas, exames de laboratório e diagnósticos do paciente estejam interligados e disponíveis para os profissionais cadastrados e que prestam cuidados. Dessa forma, constata-se que a decisão clínica do EPA é sempre baseada no histórico de saúde do paciente. Com isso, é possível observar a real situação de otimização de recursos, pois não se repetem gastos desnecessários, devido à ausência de informações, e a implementação do tratamento se dá após a análise do qual foi mais efetivo.

### Unidade Hospitalar

A visita no UF *Health Shands Hospital* iniciou na Unidade de Terapia Intensiva cardiológica pediátrica e depois no Centro Cirúrgico. Em ambos os locais, há presença e atuação de EPAs e enfermeiros assistenciais. Eles e vários outros profissionais da saúde trabalham em equipe. Em geral, os EPAs fazem as avaliações dos pacientes, solicitam e interpretam exames, fazem as prescrições medicamentosas e prestam cuidados avançados de alta complexidade.

Visitar esses serviços, nos distintos níveis de atenção, contribuiu para o grupo na compreensão do papel do EPA no serviço de saúde, assim como na percepção das diferenças entre o EPA e os demais enfermeiros.

### Research Summit: Conhecendo os Projetos de Pesquisa dos Enfermeiros de Prática Avançada

A pesquisa e a inovação em saúde também foram destaques durante a semana de imersão. No último dia da atividade, ocorreu o “*Nursing Research Summit 2024*’, promovido e realizado na Escola de Enfermagem da UF. O evento debateu inovações na aplicação de Inteligência Artificial (IA) e soluções móveis baseadas em evidências no cuidado de enfermagem, com o tema “Inteligência Artificial e Soluções de Saúde em Enfermagem: Otimizando o Atendimento ao Paciente”.

Neste evento, foi realizada a exposição de estudos científicos e de melhoria de qualidade para a prática profissional da enfermagem, e em especial para a PAE, com diversos temas inovadores, tais como os fatores de proteção e risco na enfermagem e a capacitação de novos enfermeiros em pesquisa prática baseada em evidências. Foi evidenciada uma ampla amostra de estudos recentes, revelando o impacto e a credibilidade crescente da UF na pesquisa em enfermagem. A colaboração entre enfermeiros, cientistas de dados e gestores de saúde foi destacada como essencial para desenvolver soluções que se alinhem com as necessidades específicas da prática de enfermagem e do cuidado ao paciente. O evento também contou com dois painéis de debate sobre educação e simulação em enfermagem e soluções de saúde com uso da IA, além da exposição de 83 estudos científicos e de melhoria de qualidade na modalidade de pôsteres.

Esses *insights* compartilhados entre profissionais da área ampliam nosso entendimento sobre o impacto das tecnologias emergentes na enfermagem e também inspiram uma reflexão contínua sobre como integrar as novas ferramentas de maneira ética e eficaz na prática profissional.

## DISCUSSÃO

Embora os sistemas de saúde americano e brasileiro sejam bem diferenciados, um sistema fundamentado em seguros, e outro um sistema universal e de bem-estar social, essa imersão propiciou um entendimento do papel do EPA naquele país, oferecendo uma visualização de como esse profissional (no Brasil) pode receber uma formação para um cuidado clínico a nível avançado. Especialmente na Atenção Primária à Saúde, NPs fazem a diferença naquele país, promovendo acesso àqueles que ficam à margem do sistema e sendo resolutivos no cuidado oferecido, reconhecidos como os provedores primários de cuidado na comunidade em que atuam.

O modelo de cuidado do NP se mostrou similar ao desempenhado pelos enfermeiros brasileiros na atenção primária, especificamente aqueles formados pela pós-graduação *lato sensu* em saúde da família, cujo enfoque tem sido a prevenção e promoção da saúde, mas também o cuidado, a reabilitação, a liderança e a gestão de casos complexos na rede de cuidados de forma integral e enfocando na determinação social do processo saúde-adoecimento dos indivíduos e comunidades. Entretanto, o que diferencia ambos os profissionais é a prática clínica ampliada, que garante ao enfermeiro autonomia para o cuidado clínico, diferente da realidade brasileira, pois os enfermeiros não dispõem da autoridade para: prescrição de todos os medicamentos que exijam a necessidade de prescrição, independente de controle médico; solicitação de exames médicos laboratoriais, de imagem e dispositivos (sem estar vinculado a protocolos e cuidado); decisão e indicação de tratamentos médicos e terapias; realização de diagnóstico clínico diferencial, de efeitos colaterais, estadiamento de doenças, ou avaliações avançadas de saúde; responsabilização pelo cuidado clínico sobre um conjunto (carteira) de usuários; fazer referência e contrarreferência de usuários na rede; e ser reconhecido como o primeiro ponto de contato na unidade de saúde e pela rede de atenção^(2)^.

Nos EUA, os EPAs atuando na Atenção Primária à Saúde representam 88% desses profissionais e 73,3% na saúde da família. Realizam um bilhão de atendimentos ao ano e prescrevem medicamentos em todos os estados americanos. Um aumento na população em geral, o envelhecimento dos *baby boomers* e o número de indivíduos vivendo com doenças crônicas impulsionarão a necessidade de acesso robusto aos cuidados primários. Os NPs são considerados uma solução eficiente para cuidados de saúde acessíveis e centrados no paciente^(5)^.

No Brasil, a enfermagem representa 57% da forma de trabalho em saúde. Entre os profissionais de enfermagem, 25% são enfermeiros e, dentre esses, 72% atuam na Saúde da Família e 15% na da Atenção Básica^(10)^. A formação e o reconhecimento do EPA no Brasil podem mudar a realidade de saúde do país, influenciando o cuidado centrado no paciente e na população, e as políticas públicas que melhorem o acesso e a resolutividade do cuidado nesse âmbito de atenção. Ressalta-se que o EPA teria mais preparo para melhorar os sistemas e a políticas de saúde, a exemplo do que ocorreu nas realidades em que foi implantada^(11)^. A formação e a adoção do EPA têm sido uma tendência global, desde os anos 2000, para atender às disparidades mundiais de saúde^(12)^.

Pondera-se sobre o incentivo à necessária mudança na formação acadêmica dos enfermeiros brasileiros com ênfase na prática clínica. Para tanto, é necessário que as escolas compreendam que a prática avançada se faz com conhecimento clínico avançado e assumam que um investimento na capacitação é crucial^(13)^. Deve-se considerar que o EPA deve ter competência de raciocínio e avaliação clínica avançada para tomada de decisão baseada em evidências de efeito e resultado ágil e preciso. A PAE confirma ser uma estratégia primordial para transformar a saúde da comunidade, e a experiência vivida revelou a importância desses profissionais no cenário americano, com impacto no acesso e resolutividade do cuidado, além da forte atuação na promoção da saúde e na prevenção de doenças.

## CONCLUSÃO

A participação dos profissionais brasileiros na semana de imersão na UF foi uma experiência enriquecedora, pela convergência entre a implementação de pesquisa, inovação e PAE. Destaca-se o potencial do EPA de proporcionar o direito à saúde principalmente àqueles em distantes espaços rurais e urbanos remotos. Esse profissional pode impactar o direito à saúde, que ainda é muito desigual em nosso país.
